# Familial chilblain lupus caused by an activating mutation in STING

**DOI:** 10.1186/1546-0096-13-S1-O62

**Published:** 2015-09-28

**Authors:** N König, C Fiehn, H-M Lorenz, MA Lee-Kirsch

**Affiliations:** 1 Technische Universität Dresden, Molekulare Pädiatrie, Klinik für Kinder- und Jugendmedizin, Dresden, Germany; 2 ACURA-Rheumazentrum, Baden-Baden, Germany; 3 Universität Heidelberg, Sektion Rheumatologie, Medizinische Klinik V, Heidelberg, Germany

## 

Familial chilblain lupus is a monogenic form of cutaneous lupus erythematosus characterized by cold-induced cutaneous lesions at acral location. It is caused by loss-of-function mutations in the nucleic acid metabolizing enzymes TREX1 or SAMHD1. Gain-of-function mutations in STING (stimulator of Interferon genes) have been described in an infancy-onset autoinflammatory syndrome with fever, inflammatory cutaneous lesions and interstitial lung disease.

Here we report on a family with dominant chilblain lupus over 4 generations. Affected family members presented with acral inflammatory and partially necrotizing lesions beginning in early childhood. In some cases, low-titered ANAs and immune complexes were detectable. The family tested negative for TREX1 or SAMHD1 mutations. Exome sequencing revealed a heterozygous STING mutation segregating with chilblain lupus in the family. The mutation affects a highly conserved residue within the STING dimer interface and is predicted to be pathogenic. Quantitative RT-PCR analysis showed an increased expression of IFN-stimulated genes in blood cells of affected family members suggesting that the identified mutation has an activating effect on type I IFN signaling. Taken together, our findings demonstrate that gain-of-function mutations in STING can cause familial chilblain lupus and expand the spectrum of type I interferonopathies.

